# A systematic literature review on the utilization of extended operating room hours to reduce surgical backlogs

**DOI:** 10.3389/fpubh.2023.1118072

**Published:** 2023-04-13

**Authors:** Mariana Oliveira, Valérie Bélanger, Angel Ruiz, Daniel Santos

**Affiliations:** ^1^CEGIST, Instituto Superior Técnico, Universidade de Lisboa, Lisbon, Portugal; ^2^Department of Logistics and Operations Management, HEC Montreal, Montreal, QC, Canada; ^3^Interuniversity Research Centre on Enterprise Networks, Logistics and Transportation (CIRRELT), Quebec City, QC, Canada; ^4^Faculty of Business Administration, Université Laval, Quebec City, QC, Canada

**Keywords:** healthcare, hospital management, operating room planning and scheduling, elective patients, surgical backlogs, extended hours

## Abstract

This article is part of the Research Topic ‘Health Systems Recovery in the Context of COVID-19 and Protracted Conflict'

**Introduction:**

Hospital managers address elective patient surgical backlogs with different strategies: increasing installed capacity, managing demand and improving efficiency. Recently, and particularly since the COVID-19 elective surgery suspension, extended operating room hours has been used to reduce waiting lists by taking advantage of empty operating rooms and existing surgical teams.

**Methods:**

Two research questions are raised: (1) which are the scientific literature's insights related to the use of extended operating room hours to help reduce surgery backlogs? and (2) provided that a hospital decides to extend its operating room opening time, what are the main challenges and the key aspects to consider in the design and implementation of policies to manage extended operating room hours? A systematic review on Web of Science database was conducted to gather existing literature, published from January 2012 to December 2021, regarding strategies to reduce waiting lists using empty operating rooms outside regular working hours.

**Results:**

A total of 12 papers were selected as relevant to address the two research questions. Results were organized according to their main features, namely setting, type of strategy, methodology, and how human resources are handled.

**Discussion:**

The review suggests that extended operating room hours might be problematic if current staff is used and that a careful choice of patients should be made. However, its potential to reduce waiting times and its implications are discussed only superficially. Therefore, we analyze the implications of extending operating room hours from four different perspectives (management, staff, patients, and strategy deployment) and define some recommendations for policy makers and healthcare managers when implementing it in practice.

## 1. Introduction

Long waiting lists for surgical care are a significant problem with an impact on patient satisfaction and outcomes. In OECD countries, patients can wait up to several months to have their surgery performed (1). For instance, in Portugal, by the end of 2019, over 27,000 patients on waiting lists had been waiting for more than one year to receive surgery (2). The COVID-19 pandemic has drastically worsened the situation as most hospitals had to cancel or reduce elective surgeries. In the United States, Berlin et al. (3) report a decrease of around 35% in the number of surgeries performed between March and July 2020, when compared to the same period in 2019, and estimate that hospitals in the United States would need to work at 120% of their historical throughput during ten months to be able to work through two months of additional surgical demand in <1 year. Aldecoa et al. (4) estimate that the number of elective surgeries that were canceled or postponed worldwide during the 12-week period of disruption in spring 2020 amounts to 28,404,600. As a consequence, health organizations face considerable surgery backlogs. This is an issue that deserves attention due to their strong impact on the health system and the patients (5). In order to guarantee timely access to care, innovative and systematic approaches must be considered.

Broadly speaking, three approaches can be adopted to increase surgical activity: increase the installed capacity (infrastructure, human resources, and equipment), manage demand (for example, by offering alternative treatments), and improve efficiency. A straightforward manner to reduce waiting lists is by increasing capacity by constructing new operating rooms (OR) or training additional staff. However, this may take several years (6), thus, it is a strategy that can only be used as a long-term one. OR capacity can also be increased by improving practices, either through maximizing OR business hours utilization or minimizing idle times (7–9), or by reducing surgery lengths, for example, using more advanced procedures or more sophisticated equipment. Finally, financial incentives (1) have also led to increases in surgery productivity, although the reported improvements varied considerably between hospitals (10).

This paper is devoted to a fourth approach that differs in a subtle yet profound manner from the previous ones. The approach, which will be referred to as extended OR hours, has been observed in some hospitals and consists, essentially, of extending the use of OR beyond their regular opening hours to allow the existing surgical teams to increase their production. OR are usually open for a maximum of 10 h a day and, in most cases, are idle and not staffed for the remaining 14 h (except for emergency patients) (11). Additionally, given the availability of resources or due to cancellations or other unforeseen events, not all regular open hours of OR defined in the master surgery schedule (MSS) are effectively used for surgery. For instance, hospitals in Portugal and Spain receive an extra payment from their national healthcare systems for each surgery performed outside the regular working hours of OR (1). A part of this extra payment is then distributed among the members of the team performing the surgery. Indeed, extended OR hours increase the funding dedicated to surgical activities without increasing the installed capacity, since hospitals use the already available resources (OR and staff) to perform more surgeries. Extended OR hours should not be confused with classical extra-time management, which refers to cases where a surgery scheduled in regular time finishes later than the closing time of OR, either because it took longer than expected or because its starting time was delayed. Moreover, in extended OR hours, the surgical teams are not forced but rather invited to work additional hours on a voluntary basis, which means that hospitals need to negotiate and stimulate their participation.

Surprisingly, despite some basic administrative information concerning the funding procedures associated with the payment of the surgeries carried out outside regular hours of OR, we were not able to find rules or guidelines framing the use of extended OR hours with respect to how to decide the number, the time and the profile of the specific surgeries to perform in extended time. Furthermore, we did not find any document linking or coordinating the planning of surgeries to perform in extended OR hours to the master surgery schedule (MSS) plan that establishes the regular OR production. Considering the utter need for increasing the volume of surgeries, we believe that hospitals worldwide are, or will be soon, considering similar approaches to cope with such never seen before backlogs and, consequently, face the challenges and lack of guidelines to implement them.

Extended OR hours are a possible solution to these backlogs, given that they seek to achieve a temporary capacity increase by bringing into play existing staff and OR resources outside the regular working hours. In the short term, the benefits of performing surgeries in extended OR hours are straightforward: more surgeries, staff premiums, and higher hospital income. However, if the problem of elective surgical patient backlogs is structural, the rightness of extended OR hours production must be carefully assessed, since the implementation of such strategies requires the agreement of the OR staff and affects the rest of the human resources of the hospital and, clearly, the patients.

In fact, OR staff involvement is a necessary condition for extended OR hours to be used as a strategy. Lovejoy and Li (11) discussed hospital negotiations with surgical staff concerning evening work. Surgeons and staff avoid work during these hours because of two reasons: quality-of-life considerations and accumulated delays during the day. The authors conclude that mitigating the uncertainty at the beginning of the period from which the OR might be available for extended hours, alongside bonus payments, might reduce resistance to fill in OR extended hours. For this reason, we can conclude that the use of extended OR hours requires adequate planning.

The literature on OR planning is comprised of many studies focused on a large spectrum of decisions, such as the case-mix (i.e., the volume and properties of the surgical services to be offered by a hospital), the capacity to be installed in the hospital (namely how many OR and their staffing), the assignment of the available capacity to surgical specialties or groups of surgeons (also known as the master surgery schedule, MSS), the assignment of patients to specific OR sessions, and the scheduling of surgeries within sessions (12). However, these studies concern a regular activity context. Extended OR hours go beyond the standard OR planning and scheduling framework, raising specific questions and challenges, and it has not been, to the best of our knowledge, formalized and treated before in the literature.

At this point, it is important to reiterate that this concept should not be mistaken with related concepts such as flexible contracts, open scheduling, modified block scheduling, or overtime. Furthermore, as healthcare systems operate differently from one country to another, and due to the interdependence of the resources involved in OR planning, it is unrealistic to expect a single solution to explore extended OR hours that fit all the cases. Therefore, it is important to review and consider all the different perspectives and application contexts concerning the literature with respect to the use of extended OR hours before considering its deployment.

In this context, this paper raises the two following main research questions: (1) which are the scientific literature's insights related to the use of extended operating room hours to help reduce surgery backlogs?; and (2) provided that a hospital decides to extend its OR opening time, what are the main challenges and the key aspects to consider in the design and implementation of policies to manage extended OR hours? To answer, at least partially, these broad yet important questions, this paper develops a systematic review of the literature devoted to OR capacity planning with a focus on contexts where surgeries can be scheduled outside of the regular working hours. The works reported by the search will then be used to understand the implications of increasing OR hours as a strategy to reduce surgical backlogs. This analysis should raise discussions, both at the hospital and the national levels, on how to manage this potential avenue to expand surgical capacity. To the best of our knowledge, this is the first work that reviews the scientific literature on OR planning with the aim of formalizing the use of the idle OR capacity.

The remainder of this paper is structured as follows. Section 2 describes the methodology used to identify the scientific works related to extended OR hours, and Section 3 discusses the main findings of the literature review. Section 4 discusses important considerations regarding the management of extended OR hours and establishes the main practice implications of implementing such a strategy.

## 2. Methods

This section describes the methodology applied to search the scientific literature and to identify the contributions that include any strategy, model or case that aims to reduce surgical waiting lists by using empty OR outside their regular working hours to perform additional surgeries. As mentioned previously, such strategies are referred to as extended OR hours in this paper. A preliminary search of recent literature reviews devoted to OR planning and scheduling confirmed the lack of approaches related to extended OR hours (13–15).

On December 10, 2021, a systematic search was conducted on the Web of Science database with the following query:

(TS=(“healthcare”) OR TS=(“health care”) OR TS=(“care”) OR TS=(“operating room*”) OR TS=(“operating theat*”) OR TS=(“surg*”)) AND (TS=(“backlog”) OR TS=(“temporary capacity change”) OR TS=(“max* capacity”) OR TS=(“improve* use of existing capacity”) OR TS=(“capacity expan*”) OR TS=(“expand* capacity”) OR TS=(“increas* capacity”) OR TS=(“extra capacity”) OR TS=(“extra time”) OR TS=(“extratime”) OR TS=(“overtime”) OR TS=(“after-hours”) OR TS=(“after hours”) OR TS=(“outside work* hour*”) OR TS=(“extend* hour*”))

**Figure 1:**
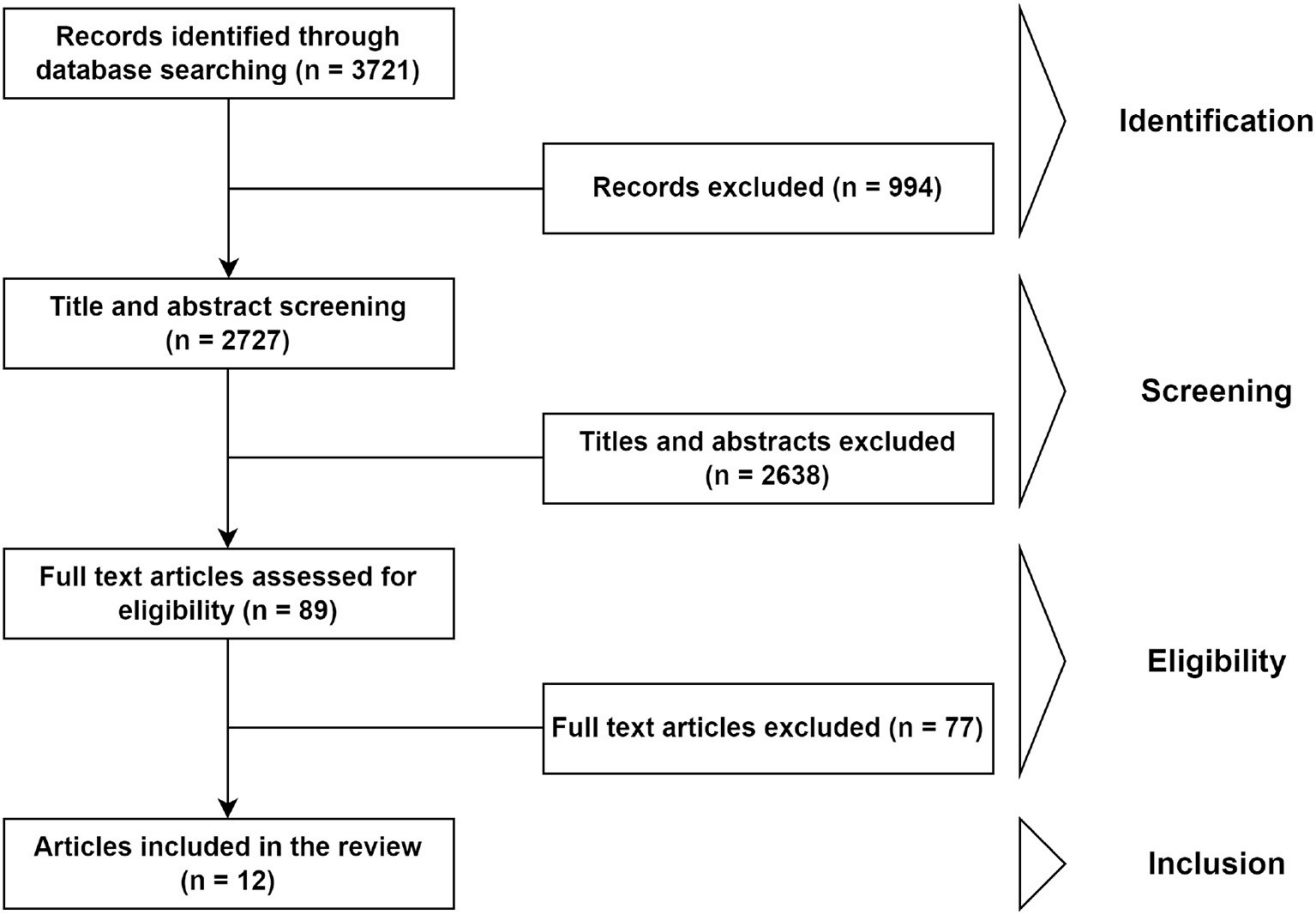
Document selection scheme used for the systematic review of the literature in the Web of Science database.

This search retrieved 3,721 results, of which 3,511 were written in English. Of those, we decided to exclude papers published before 2011, resulting in a final pool of 2,727 papers for analysis. Any paper mentioning planning strategies that involve either temporary change of service capacity or increasing working hours or flexibility of human resources to proactively increase productivity and reduce backlogs in healthcare by taking advantage of extended time was considered potentially relevant. We included not only surgical settings, but also non-surgical settings that operate in a similar way to the former, to increase the potential for finding extended time strategies. However, documents focused on emergency services were excluded on account of their 24/7 nature, which makes any separation between regular and extended hours irrelevant. No exclusion criteria regarding the quality or the type of document were applied. The document selection scheme is summarized in Figure 1.

By screening titles and abstracts, the pool of documents was reduced to 89 papers. During this analysis, several research trends or topics were identified: impact of after-hours surgery or admission (mostly focused on trauma or emergency services), open scheduling, or extended opening for temporal convenience (usually associated with increasing business hours to reduce the number of people in emergency services). These research topics were discarded for the present literature review because they do not fit the extended OR hours scope. However, they contribute interesting discussions on the implications of introducing flexibility in schedules through the increase of working hours that, in our opinion, also apply to the extended OR hours context. After reading the 89 full documents, 12 papers were selected to be relevant as a result of this literature search.

## 3. Results

Tables 1, 2 summarize the main features and the contributions of the 12 papers resulting from the search described in the previous section. These tables include, for each paper, their Methodology (Metho.) and their setting, which encompasses the country (Count.), if the application was developed before or after the Covid-19 pandemic (Pre/Post), and the clinical specialty or service (Serv.). Then, the four next columns characterize the type of strategy it proposes. The first two of them are related to Increase Capacity (Inc. cap.), which includes Extended Hours (E.H.) and other approaches (Other), and the two following columns characterize Manage demand (Man. Dem.) and Increase Productivity (Incr. Prod.). The last column describes the paper's objective. Table 1 presents papers related to non-surgical settings, whereas Table 2 reports studies on surgical settings. These settings differ mostly on the resources required, which can, in turn, impact the strategy proposed. Surgical settings typically require specialized equipment along with a multidisciplinary team, which is not necessarily the case in other settings. Moreover, decisions on surgical settings extend beyond the OR, affecting, for instance, intensive care units and hospitalization units.

**Table 1:** Resulting papers concerning extended hours in non-surgical settings.

					**Strategies**				
		**Context**			**Inc. cap**.				
**Reference**	**Metho**.	**Count**.	**Pre/post**	**Serv**.	**E.H**.	**Other**	**Man. Dem**.	**Incr. Prod**.	**Objective**
Kantarevic et al. (16)	DA	CA	Pre	OC	ES			EFFS	Compare enhanced FFS to standard FFS models
Tan et al. (17)	LR, SUR	SI	Pre	OC	AS		T		Evaluate impact of several measures on waiting time
Fagefors et al. (18)	DA, LR, SUR	SW	Pre	OC, HNSU	ES	OT, EP	T		Study creation of short term capacity flexibility in healthcare systems
Azam et al. (19)	LR	US	Post	IM	ES		T	AP	Review guidelines and literature to help resuming non-urgent imaging services

Methodology (Metho.): DA, Data analysis; LR, Literature review; SUR, Survey / Expert opinion.

Country (Count.): CA, Canada; SI, Singapore; SW, Sweden; US, United States.

Service (Serv.): OC, Outpatient clinic/primary care; HNSU, Hospital non-surgical unit; IM, Imaging.

Strategies: ES, Existing staff; AS, Added staff; OT, Overtime; EP, External providers; T, Triage/Prioritization; EFFS, Enhanced fee-for-service; AP, Abbreviated protocols.

**Table 2:** Resulting papers concerning extended hours in surgical settings.

					**Strategies**				
		**Context**			**Inc. cap**.				
**Reference**	**Metho**.	**Count**.	**Pre/post**	**Serv**.	**E.H**.	**Other**	**Man. Dem**.	**Incr. Prod**.	**Objective**
Bhuiyan and Mavhungu (20)	DA	SA	Pre	GEN	ES				Determine the cost and death rate for weekend surgeries
Lin and Chou (21)	DP	UK	Post	CAT	U	EP	T, PST		Discuss the improvement of services post-pandemic
Macdonald et al. (22)	DA	UK	Post	GEN	ES, AS	EP	PT		Forecast backlogs and propose solutions to prevent a public health crisis
Moawad et al. (23)	DP	US	Post	GEN	U		T		Highlight challenges of returning to normality
Samson (24)	DP	CA	Post	NA	AS				Discuss solutions to eliminate backlogs while maintaining regular throughput
Anastasio et al. (25)	DP	US	Post	ORT	ES				Discuss after-hours teams and protocols to improve efficiency
Clifford et al. (26)	DA	UK	Post	LC	U				Analyze dedicated operating lists to reduce waiting lists
Barie et al. (27)	LR, SUR	US	Post	GEN	U		T, PT		Give guidance for safe and effective resumption of surgeries

Methodology (Metho.): DA, Data analysis; DP, Discussion paper; LR, Literature review; SUR, Survey / Expert opinion.

Country (Count.): SA, South Africa; UK, United Kingdom; US, United States; CA, Canada.

Service (Serv.): GEN, General; CAT, Cataract; ORT, Orthopedic; LC, Laparoscopic cholecystectomy; NA, Not available.

Strategies: ES, Existing staff; AS, Added staff; U, Unknown staffing; EP, External providers; T, Triage/Prioritization; PST, Postponement; PT, Patient transfer.

Among the 12 papers, we observed a variety of propositions that we grouped into three families of strategies: (1) strategies that seek to increase the opening hours of the corresponding service, including weekend and after-hour clinics; (2) strategies to better manage demand, including prioritization and triage, treatment postponement, and patient transfer; and (3) strategies to increase productivity, such as specific fee-for-services to reward physicians and reduced protocols. It is worth noting that several of the papers were published after March 2020, therefore after the beginning of the COVID-19 pandemic. These papers discuss strategies to resume services after lockdown. While the COVID-19 pandemic exacerbated waiting lists, we assume that similar strategies can be envisioned to address backlogs regardless of the situation (pre- or post-pandemic). Nevertheless, the volume of surgeries or services to be performed can be larger in a post-pandemic situation, and specific infection control methods can impact the time to provide service. Therefore, we deemed it important to identify if the study considered a pre- or post-COVID setting.

Table 1 contains four studies that analyze and discuss strategies to reduce waiting lists in non-surgical settings. These studies concern a variety of contexts in different countries, including primary care (16, 18), outpatient clinics (17, 18), hospital units (18), and imaging and radiology (19), and were conducted mostly before the COVID-19 pandemic (16–18). We observed that pre-COVID studies tend to perform deeper analysis and evaluations compared to post-COVID studies, in particular through data analysis. In the next paragraph, we present a detailed description of these studies, focusing on their objectives and key findings.

In 2011, Kantarevic et al. (16) described an enhanced fee-for-service model that encourages providers to improve patient access and quality of care through contractual extended service hours (evenings, weekends, and holidays) in the context of primary care in Canada. The enhanced fee-for-service model aims to increase productivity by rewarding doctors. Using an economic model, they showed that this new payment model increased primary care physician productivity, measured as the number of services provided and patients treated compared to typical fee-for-service reimbursement. Overall, the authors estimated productivity gains of 6–10%, corresponding to 2–3 weeks of additional work over a year.

Tan et al. (17) described a case study in which a working group was created to find ways to reduce long waiting times in the General Pediatric Clinic in Singapore in 2017. The implementation of weekend clinics to clear backlogs of outpatient referrals was one of the initiatives proposed by the group. This initiative targeted specifically patients with a waiting time longer than 60 days. The implementation of weekend clinics, together with other improvements such as, for example, a triage system, allowed the General Pediatric Clinic to reduce waiting times by 30%. The authors concluded that weekend clinics appear to be effective in reducing waiting times, but, at the same time, they highlighted the lack of existing guidelines on when to open and how to manage those extra clinics.

Fagefors et al. (18) conducted a survey in which Swedish healthcare managers were asked about the strategies they used to create short-term volume flexibility in healthcare capacity to cope with demand fluctuations. The study concerned a wide range of hospital units, including non-surgical and surgical units. Among the reported conclusions, the authors emphasize that managers preferred to plan fixed overtime rather than use it as a response to specific situations and use on-call staff to fill the schedule gaps. The authors also underline that the type of strategies to use depends on the type of care unit and services it offers (e.g., acute care or not).

Like many post-pandemic studies, Azam et al. (19) provided an open discussion based on the review of guidelines rather than a formal evaluation of strategies to develop a roadmap for radiology departments with the aim to resume non-urgent imaging studies and elective procedures. In particular, they stress the importance of taking into account the context raised by COVID-19 to adapt practices, including specific infection control measures. However, to reduce backlogs, they suggested relying on extended hours of operation and extended shifts for radiologists. They highlighted that the implementation of such practices should be staged, incremental and regularly reviewed, and, at the same time, communication and transparency are required.

Table 2 presents the eight studies grounded in surgical settings. These studies considered various types of elective surgical settings, including general surgery, cataract, orthopedic, and laparoscopic cholecystectomy, and all but one were conducted in the UK and the US after the COVID-19 pandemic. In addition to the families of strategies presented, these papers also addressed several COVID-related measures, such as dedicated COVID-19 facilities and supplying of personal protective equipment. However, these measures are not discussed in the ensuing text since they are unrelated to extended OR hours. It is possible to observe that few of the papers include data analysis with a specific objective. In fact, most papers would rather include a discussion on ways to return to normal operations after COVID-19. In the next paragraphs, we first discuss papers that provide data analysis and then highlight key intakes from discussion papers.

Bhuiyan and Mavhungu (20) is the only paper in a surgical setting published before the COVID-19 pandemic. In this paper, the authors recommended Saturday elective surgeries for a hospital in South Africa as a course to maximize the use of OR in all tertiary hospitals that do not have a daytime emergency room or/and long waiting times. This study assessed the quality of surgeries performed on Saturday—by computing the death rate within 30 days for patients having received their surgery on Saturday—as well as the cost of performing those surgeries. Based on a prospective observational descriptive cohort study, it concluded that surgeries performed on Saturday have an associated death rate of 1.5% (compared to 1.9% on weekdays) and an additional cost of between ZAR 2,317 to 3,450 per patient to cover overtime costs. However, the authors suggested that high-risk patients should have their surgery performed on weekdays. Saturday patients, therefore, need to be selected carefully, confirming the effectiveness of extended OR hours, but raising a key question on how these should be used.

Using data published by NHS Digital and NHS England, Macdonald et al. (22) estimated the number of cancellations and missed cases of elective surgeries due to COVID-19. The authors concluded that surgery backlogs were rising even before the COVID-19 pandemic. They also stated that weekend working is well-defined in the National Health System, and it has been a successful temporary strategy that, although requiring additional funds, is able to reduce waiting times. Moreover, it is also recommended that adding a third session to the OR can constitute an option to replace the capacity loss due to time-consuming COVID-19 safety protocols.

Clifford et al. (26) analyzed data on financial implications, demographics, procedures and outcomes after and before the implementation of five intensive dedicated operating lists (i.e., waiting lists deliberately created to address backlogs) for laparoscopic cholecystectomies on weekends in October 2020 at a single Trust. The analysis demonstrated the benefits of performing weekend surgeries, rapidly reducing waiting lists for laparoscopic cholecystectomy elective surgery after COVID-19. However, the authors recommended a careful selection of patients, which should be done by a multidisciplinary team.

Lin and Chou (21) proposed care providers to develop a new patient-centered clinical pathway by redesigning the cataract service in the post-COVID-19 era. One of the proposals they discussed is the possibility of considering routine cataract integrated practice units with high volume weekend/evening following independent patient lists. Moawad et al. (23) presented a perspective paper proposing to extend surgical hours on weekdays to increase OR access to resume elective surgical activity after the COVID-19 suspensions. In a letter to the editor, Samson (24) quantified the amount of additional OR hours, or the required extra shifts to be added to daily schedules, to compensate for the cancellation of cases due to the COVID-19 pandemic. The author stressed the fact that overtime undermines the performance of OR staff, encouraging managers to be prudent on its usage. In addition, it is suggested to rely on retired medical professionals to increase regular staff, and, finally, it is recommended to allow additional 8-hour OR shifts in the evenings or on weekends. Anastasio et al. (25) wrote a commentary note suggesting after-hours surgery as a timely and cost-effective strategy to deal with backlogs due to the COVID-19 shutdown, but also alerted to the potential consequences of staff exhaustion. To mitigate this, it was suggested to form dedicated nighttime surgical teams, ensuring them rest time during the day.

Finally, Barie et al. (27) reviewed and summarized peer-reviewed literature, while integrating it with expert opinion on guidelines for resuming surgical services during the COVID-19 pandemic, to improve outcomes and safety, preserve resources, reduce costs and ultimately reduce surgical backlogs. Besides key points, such as institutional commitment, structural programs, equitable implementation, audits, and planning and integration, the authors stressed that case prioritization during the resumption of services needs to consider surgical risk factors, COVID-19-related risks and facility capacity, through the maximization of the capacity of outpatient facilities and the use of off-hours.

The utilization of unused OR time, such as after-hours or weekends, to increase capacity and, eventually, reduce backlogs, has been suggested in the literature. However, while interest in this type of strategy has grown as a result of the worldwide suspension of elective surgeries during COVID-19, its potential in reducing waiting lists has only been studied superficially. Indeed, there is a lack of models, case studies, and guidelines that might help managers develop and implement extended OR hours strategies at a time when they might make a difference. Moreover, during the development of this literature review, a gap was evident in terms of the discussion of key decisions in such a context and their impact on patients, staff, and the health system in general. Nevertheless, two main insights come from those papers. First, they acknowledge that extended hours, either after-hours or weekends, could be problematic if current staff is used, leading to fatigue, overload, and burden. Therefore, we need to find ways to recruit staff (e.g., retired clinicians, having dedicated staff, etc.). Second, patients to be operated on need to be chosen carefully. These issues and other questions that we deemed central to the deployment of extended OR hours strategies are discussed in the next section.

## 4. Discussion

The pressure on health systems worldwide to deliver appropriate care has never been so high. In the case of surgical services, the reduction of activity imposed by the COVID-19 pandemic exacerbated an already existing problem of extensive backlogs, which result in a drastic deterioration of the access time to surgeries. We have identified two main goals inspiring the reviewed research papers: resuming activity post-COVID (i.e., defining guidelines for isolation pathways, safety protocols, prioritization or treatment simplification), and increasing capacity or extending service hours, although no study thoroughly assesses their impact and implications.

We believe that both goals are strongly affected by the fact that human resources are even scarcer than before the pandemic, given that the entire health system is exhausted by this fight and OR staff have been deployed to reinforce services in deeper need. Therefore, recovering the pre-pandemic staffing levels seems rather optimistic, at least in the short term. Meanwhile, managers must carefully analyze the extent to which backlogs have been originated by structural (permanent offer to demand unbalance) or transient (the pandemic) reasons to decide on the set of actions to take.

To shed some light on the complex decisions related to extended OR hours, the next subsections review some crucial aspects, grouped into four categories: the management, the staff, the patients, and the deployment of the strategy. We believe these are the aspects managers should ponder when formalizing their extended OR hours strategy.

### 4.1. The management

Extended OR hours may allow hospitals to increase their financing and/or avoid penalties, in case their funding is tied to performance targets. However, additional surgeries also require additional costs, thus, their profitability needs to be assessed and the contribution margin must be considered in the specific situation of each hospital. Sutherland (28) described the case of hospitals in British Columbia (Canada) that received financial incentives to increase surgical volume through marginal pricing approaches and analyzed four scenarios that represent situations with decreasing workforce availability. The first scenario corresponded to staff overcapacity, so the available staff is able to perform more surgeries. In the second scenario, staffing was adequate, but it was assumed that improvements in efficiency allowed to increase the number of performed surgeries. The remaining scenarios considered undercapacity: in the third scenario, the hospital used overtime to increase the number of surgeries, whereas in the fourth scenario premium rates were paid. Sutherland (28) concluded that, in order to be efficient, strategies and specific financial incentives need to be adjusted according to the underlying cost structures of the hospital. Specifically, since some surgical cases are more profitable than others, it is important to establish guidelines and mechanisms that ensure that the economic criterion is not the only one considered in allocating the extra time to specialties. In the same vein, managers must be aware that the incentives and rewards associated with activities in extended OR hours might create competition between the hospital services or departments.

### 4.2. The staff

Extended OR hours require a voluntary engagement of staff, who offer to work on specific days and/or time slots which are outside their regular working hours. Therefore, staff needs to be adequately compensated or rewarded for their additional effort. Rewards for extra work typically include extra time off, promotion, recognition and financial rewards (29). However, Broadway et al. (30) showed that economic rewards alone are ineffective in promoting engagement in after-hours work and may even be harmful if incentives are not properly targeted. Almaghrabi et al. (31) conducted a web-based survey to healthcare workers to assess their willingness to work overtime and extra hours during the COVID-19 pandemic. The results demonstrated a high positive response and engagement, confirming that, when the situation calls for it, health workers are open to working out of their regular schedules to achieve a certain goal. This result shows why the communication of the aim of the strategy is so important to its success. Note, however, that if too many workers are willing to fill in extended hours, managers must allocate the extra time in a fair and transparent manner to preserve the motivation and the engagement of the workers.

Another important aspect of extended OR hours, which has also been discussed in Section 3, is that it might have a negative impact on the workers' performance, as well as on their health, safety, and wellbeing. Lobo et al. (32) reported that nurses working overtime suffer more injury hazards and have less ability to achieve a good work-life balance, while Stimpfel et al. (33) account that newly licensed nurses working weekly overtime are associated with a higher risk of needle stick. As an advantage, voluntary overtime hours seem to reduce turnover among nurses (34). Although these papers refer to overtime and not extended OR hours, both strategies share the trait of involving surgical staff working additional hours, which might lead to reduced quality of life for these hospital workers.

### 4.3. The patients

Increasing the number of surgeries performed is clearly aligned with the needs of patients. However, throughput is not the only goal to pursue. In fact, extended OR hours strategies should be patient-centered, although some studies have shown that nurses working overtime are more task-oriented than patient-centered in the care they provide (32). Promoting teamwork and assuring that all the members are engaged in a patient safety culture should be one of the priorities in managing extended capacity since, as Cortegiani et al. (35) showed in the systematic review and meta-analysis that they performed, night and after-hours surgeries, when used to reduce waiting lists, are associated with a higher adjusted risk of mortality when compared to surgeries performed during daytime, even though the existent evidence is weak. Additionally, as mentioned in Section 3, discussing criteria for choosing patients to be scheduled on extended hours and establishing adequate work guidelines is fundamental.

### 4.4. Deploying the strategy

Planning extended OR hours can be as complex as planning regular hours. In fact, similar decisions have to be taken, although potentially for different objectives and subject to different restrictions. For instance, managers must decide how many extended hours to add and also when to add them, given the regular fixed OR hours. Decisions regarding which specialties should access the extra time or how the extra time should be shared among the specialties are also similarly important in both regular and extended OR hours. Nevertheless, extended OR hours should be more flexible than the regular hours defined by the MSS, allowing the hospital to relax some constraints that limit their decisions, such as, for instance, guaranteeing a minimum number of weekly or monthly hours to each specialty. On the other hand, the assignment of staff to extended OR hours is harder than in the case of regular hours, since the staff must voluntarily agree to work these extended hours.

To handle the above-mentioned difficulties, at least two alternative models for the implementation of extended OR hours can be envisaged: a hierarchical model where the hospital plans and manages the extended OR hours as a second MSS, and a second, more participatory, bottom-up model where each service or specialty proposes additional working hours to the hospital managers, who decide the access to extended OR hours according to the established goals and mechanisms. The latter approach offers an important advantage, since it simplifies the negotiations with the staff, who self-organize into teams to elaborate proposals for the use of extended hours.

Regardless of the model used to implement extended OR hours, we would like to stress the importance of performance measurement and monitoring, for two main reasons. First, it is imperative to ensure that the extended OR hours are successfully addressing the underlying problem to be solved, namely that of reducing the extensive backlogs of patients. Second, it is also important to guarantee that regular hours activities are not negatively impacted by extended OR hours, since the latter are only complementary to the former and, thus, should not serve as a replacement.

### 4.5. Conclusions

Based on the lessons learned from the reviewed papers and our experience and discussions with healthcare managers, we would like to propose three recommendations we deem necessary, yet insufficient, for successfully implementing extended OR hours strategies.

The first recommendation is that extended OR hours, as defined in this paper (i.e., using existing human resources), should only be used in the short term or, if used in medium to long terms, its intensity should be limited to prevent exhaustion of the OR staff and the post-surgical services, which might be overloaded.

The second recommendation stresses the need for a shared understanding and agreement of all the stakeholders on the goals pursued by this strategy: is it to broaden access to services?, is it to cope with emergency surgeries?, is it to perform more surgeries of a particular type?, or is it to treat specific patients under specific situations? Only after all the stakeholders (managers, staff, and patients) have agreed on the objectives, it is possible to start framing and planning how to make the best use of extended OR hours.

The third recommendation concerns communication and transparency. Decisions should be clear and communicated to all stakeholders. This level of transparency allows decisions to be revised in light of information held by different people. Indeed, if decision-makers share the knowledge about the strategic goal of extended OR hours, it should be easier to guarantee an efficient and fair use of resources.

Although the future trends of research on extended operating room hours can take different directions, the authors recommend exploring three specific topics that can be particularly promising for future research: enquiring on the professional's motivations and the rewards that matter to them, the development of methods to quantify the “value” of a given surgery for the organization, and the study of approach to mutualize or share hospital's capacity.

Elective surgical patient backlogs constitute a challenge for healthcare organizations and may lead to the adoption of extended operating room hours to perform additional surgeries. However, extending working hours requires understanding what motivates healthcare professionals, including surgeons, nurses and anesthetists, to accept such an arrangement. Studies that assess what may motivate healthcare professional, such as financial incentives, downtime or other work benefits, are particularly encouraged. Those results will allow healthcare managers to plan, identify and quantify rewards that maximize surgical staff's satisfaction whilst reducing the risk of burnout, which is more critical than ever in healthcare.

Decisions regarding which surgeries to perform during extended operating hours should be made based on their value, which includes but it is not limited to the associated revenue. For instance, value should consider medical risks and penalties for delayed surgeries, and eventually capture the surgery's impact on the patients' quality of life. Therefore, determining the value of each surgery is pivotal when managing extended operating room hours, and should be used by decision support systems to assist managers in the selection of surgeries to maximize total value at the time that a fair access to extended time to all the specialties and professional is guaranteed.

Furthermore, hospitals should work together as a network to offer available operating room time to other organizations or exchange surgeries to minimize delays and increase patient and stakeholders' satisfaction. This approach can help to redistribute surgical demand across healthcare facilities and reduce wait times. However, it is crucial to develop a fair and equitable system to allocate operating room time among healthcare organizations and clearly set the rules for transferring patients. Research into the feasibility and effectiveness of such a network-based approach is necessary to optimize surgical care delivery.

## Data availability statement

The original contributions presented in the study are included in the article/supplementary material, further inquiries can be directed to the corresponding author.

## Author contributions

All authors contributed equally to the research design, development, and manuscript writing. All authors contributed to the article and approved the submitted version.
